# Ion Channels in Hematopoietic and Mesenchymal Stem Cells

**DOI:** 10.1155/2012/217910

**Published:** 2012-08-05

**Authors:** Serena Pillozzi, Andrea Becchetti

**Affiliations:** ^1^Department of Experimental Pathology and Oncology, University of Florence, Viale Morgagni 50, 50134 Florence, Italy; ^2^Department of Biotechnology and Biosciences, University of Milano-Bicocca, Piazza della Scienza 2, 20126 Milano, Italy

## Abstract

Hematopoietic stem cells (HSCs) reside in bone marrow niches and give rise to hematopoietic precursor cells (HPCs). These have more restricted lineage potential and eventually differentiate into specific blood cell types. Bone marrow also contains mesenchymal stromal cells (MSCs), which present multilineage differentiation potential toward mesodermal cell types. In bone marrow niches, stem cell interaction with the extracellular matrix is mediated by integrin receptors. Ion channels regulate cell proliferation and differentiation by controlling intracellular Ca^2+^, cell volume, release of growth factors, and so forth. Although little evidence is available about the ion channel roles in true HSCs, increasing information is available about HPCs and MSCs, which present a complex pattern of K^+^ channel expression. K^+^ channels cooperate with Ca^2+^ and Cl^−^ channels in regulating calcium entry and cell volume during mitosis. Other K^+^ channels modulate the integrin-dependent interaction between leukemic progenitor cells and the niche stroma. These channels can also regulate leukemia cell interaction with MSCs, which also involves integrin receptors and affects the MSC-mediated protection from chemotherapy. Ligand-gated channels are also implicated in these processes. Nicotinic acetylcholine receptors regulate cell proliferation and migration in HSCs and MSCs and may be implicated in the harmful effects of smoking.

## 1. Introduction

In early embryos, totipotent blastomeres are defined as cells able to produce every cell type of the adult organism. During development, a progressive restriction in differentiation potency occurs, with primordial pluripotent cells being able to yield other stem cells (*self-renewal*) and originate all cell lineages except the extraembryonic ones [1]. Further restriction is observed in adult tissues, in which cell lineages comprise slowly dividing stem cells. These, besides self-renewing, divide to generate *transit amplifying cells* which undergo many mitotic cycles before differentiating into the tissue cell types. For example, neural stem cells, located in restricted regions of the brain, can differentiate into neurons, astrocytes, and oligodendrocytes. Whether the spectrum of possible differentiation outcomes depends on extrinsic regulation or is founded on the existence of heterogeneous stem cell populations is uncertain [2]. A similar pattern is thought to exist in cancer tissue, in which a few stem cells maintain the neoplastic cell population, whereas the majority of cells composing the tumor rapidly divide and display only limited self-renewal properties [3].

The contribution of ion channels and transporters to the regulation of cell proliferation and differentiation is increasingly recognized. The field has greatly expanded in the last ten years and cannot be fully discussed here. The reader is referred to several recent reviews that cover the main aspects and provide introduction to specialized literature [4–11]. Although the precise mechanisms are still debated, evidence exists about the involvement of both voltage-gated and ligand-gated channels. As a first approximation, the well-known correlation between depolarization and proliferation seems to hold in embryonic stem cells. For example, inhibition of KCNQ1 potassium channels by altered expression of the accessory *β* subunit XKCNE1 depolarizes neural crest cells in *Xenopus.* This effect is accompanied by hyperproliferation [12]. Conversely, paracrine stimulation of GABA_A_ receptors, which tends to hyperpolarize embryonic stem cells and peripheral neural crest stem cells in mice, is accompanied by inhibition of cell proliferation [13].

A cell's decision to divide or differentiate is regulated by both intracellular molecular cascades and local environmental cues. Ion channels appear often to behave as signaling pivots that coordinate these upstream and downstream signals. By governing membrane potential (*V*_*m*_) and transmembrane calcium flux, ion channels regulate the calcium signals that punctuate the mitotic cycle as well as processes such as neurite extension and exocytosis of autocrine or angiogenetic factors [8]. Moreover, transmembrane ion fluxes drive the cell volume oscillations typical of cycling and migrating cells [14, 15]. However, these examples certainly do not exhaust the range of ion channel functions in cell proliferation and differentiation and other regulatory roles may or may not depend on ion transport. For instance, the channel proteins can form macromolecular complexes with growth factor receptors, cell adhesion receptors and related proteins [16–19]. These mechanisms have been especially studied in mature and immature blood cells [16, 19, 20], and their alteration can promote neoplastic invasiveness. Because of such complexity, a simple comprehensive picture of the physiology of ion channels in cell cycle and differentiation is not available and probably cannot be reached. In the following, we discuss some of these issues as related to the hematopoietic system. Since relatively few studies have addressed ion channel physiology in adult and embryonic stem cells, we believe an ample and potentially fruitful field is offered to future research.

## 2. Hematopoietic and Mesenchymal Stem Cells: The Fundamentals

Hematopoiesis allows the lifelong turnover of blood cells. In adult mammals, small populations of hematopoietic stem cells (HSCs) reside in the bone marrow (BM). HSCs originate hematopoietic precursor cells (HPCs) with more restricted lineage potential, which eventually differentiate into specific blood cells. Permanence of a functional HSC population throughout life is guaranteed by stem cell self-renewal as well as HSC dwelling in protected *BM niches*, in which 90–95% of HSCs are maintained in a quiescent state [21, 22]. The exact proportions of different cell populations and the rate of cell cycle entry vary in rodents and humans [23]. Adult quiescent HSCs arise from actively proliferating fetal HSC [24]. These are first located in placenta and aorta-gonad-mesonephros, which are subsequently substituted by the fetal liver. Fetal HSCs initially rapidly produce red blood cells and in a second phase generate all blood cell lineages plus engrafting HSCs [21, 25]. These latter actively proliferate during development by mainly generating erythroid and myeloid lineages, to produce the blood system. In rodents, HSCs colonize BM between embryonic day 17 and postnatal day 14. Subsequently, they remain anchored to the BM niche by integrin-dependent mechanisms [26, 27].

The BM stroma is constituted by stromal cells (fibroblasts, endothelial cells, macrophages, and osteoblasts) and extracellular matrix proteins. Stroma contributes to regulate hematopoiesis through signals triggered by adhesion-dependent and soluble factors. In the adult, HSCs and HPCs mostly reside in BM, whereas they actively migrate between the hematopoietic tissues during fetal development. Integrins exert a central regulatory role, as they mediate most of the functional interactions between hematopoietic cells and the bone marrow microenvironment [26–28]. The regulated expression of specific integrin subunits and their localization in selected areas of the BM confirm that these molecules exert a variety of functions in hematopoiesis [26]. The expression of cell-matrix and cell-cell adhesion receptors in HSCs and HPCs has been extensively reviewed elsewhere [20].

Mesenchymal stromal cells (MSCs), formerly known as mesenchymal stem cells, are adult stem cells originally isolated from BM. Subsequent studies have shown that MSCs are stromal progenitors also found in other adult tissues. They are endowed with multilineage differentiation potential toward mesodermal cell lineages and extensive immunomodulatory properties. MSCs actively proliferate in some tissues, but remain quiescent in others [29]. Three main criteria have been identified by the International Society of Cellular Therapy to define MSCs: (i) adhesion to plastic, (ii) expression of specific immunophenotypic marker combinations (CD73, CD90, and CD105), accompanied by lack of expression of hematopoietic markers (CD14, CD34, and CD45) and class II major histocompatibility complex (MHC) molecules; (iii) capability of differentiating into mesodermal lineages (adipocytes, cardiomyocytes, osteoblasts, and chondrocytes) [30, 31]. All cultured MSCs tend to present these features, although some differences are observed among the MSCs from a given tissue such as expression of surface CD34 and CD54 in MSCs derived from adipose tissue. In addition, MSCs may show *in vitro*, under specific experimental conditions, some features of differentiation into tissues of endodermal and neuroectodermal lineages, such as hepatocytes, epithelia, and neurons, although their genuine differentiation into nonmesodermal cells is still matter of debate [31]. Because of their properties, MSCs have drawn considerable interest as potentially useful material for tissue engineering and cell-based therapy. Human BM is now the major MSC source for both experimental and clinical studies [32–36].

## 3. Ion Channels in HSCs and HPCs

### 3.1. K^+^ Channels and Other Channels Related to the Voltage-Gated Superfamily

To the best of our knowledge, no studies have been published about the K^+^ channel expression in *bona fide* HSCs. However, inward rectifying K^+^ currents (K_IR_) have been measured in primitive HPCs (CD34+ CD38−), after stimulation with interleukin-3 (IL-3) plus stem cell factor (SCF [37]). The term inward rectifier applies to those ion channels that tend to be more permeable to ions flowing toward the cytoplasm. However, it should be remembered that not all channels known to belong to the K_IR_ structural family (Kir subunits) display prominent inward rectification. Interestingly, measurements in HPCs showed expression of both strongly rectifying (Kir4.3) and weakly rectifying (Kir1.1) K^+^ channels. Evidence that this is necessary to generate committed progenitors *in vitro* was obtained in umbilical cord blood CD34+ CD38− cells, in which inhibiting either channel type suppresses the generation of progenitor cells stimulated by IL-3- and SCF [38]. These observation are consistent with the notion that different K^+^ channel types give distinct contributions to proliferation and differentiation. In general, the strong inward rectifiers and the background channels K_2P_ (two-pore domain K^+^ channels) seem to be mainly responsible to regulate the resting *V*_*m*_. In immature B cells, Nam et al. [39] recently identified two kinds of K_2P_ channels, namely, TREK-2 (TWIK-related K^+^ channel type 2) and TASK-2 (TWIK-related acid-sensitive K^+^ channel type 2). TASK-2 is activated by stimulation of B cells receptors (BCRs ligation) and participates in the BCR-ligation-dependent apoptosis [39]. Other types of K^+^ channel are thought to cooperate with the other membrane transport systems to regulate cell volume, secretion of paracrine factors, calcium influx, and so forth. These processes may be accompanied by oscillations in *V*_*m*_, cell volume, and intracellular calcium [8]. The whole picture can thus be very complex, as is now made clear by extensive expression studies. In CD34^+^/CD45^+^/CD133^high^ cells from peripheral blood [40], RT-PCR showed expression of K_V_1.3, K_V_7.1, Na_V_1.7, TASK 2, TALK 2 (TWIK-related alkaline pH-activated K^+^ channel type 2), TWIK 2 (tandem of pore domains in a weak inward rectifying K^+^ channel, type 2), TRPC4, 6, TRPM2,7, and TRPV2 and patch-clamp recordings identified voltage gated K^+^ currents, TASK 2-like K^+^ currents, TRPM2 currents, and TRPC6-like currents [40].

Some voltage-dependent K^+^ channels, particularly K_V_11.1 (also known as hERG1), are directly implicated in regulating the integrin-dependent cell adhesion, which is potentially important for cell physiology inside the BM marrow niches [20, 41]. In leukemic osteoclastic progenitors, hERG1 activates during cell adhesion to fibronectin. Channel activation mediates a complex regulatory network that control cell adhesion, as it increases expression of *α*V*β*3 integrin (CD51 [42]). Moreover, hERG1 is upregulated in leukemic hematopoietic cells [43, 44]. In particular, the *Kv11.1 *transcript was detected in circulating CD34+ cells stimulated to proliferate by IL-3 (interleukin 3), SCF (stem cell factor), GM-CSF (granulocyte-macrophage colony-stimulating factor), and G-CSF (granulocyte colony-stimulating factor [43]. In stimulated CD34+ cells, K_V_11.1 associates with *β*_1_ integrin, which is essential for proper bone marrow engraftment of these HPCs [19]. The *Kv11.1 *transcript was also detected in CD34+/CD38−/CD123^high^ cells, which constitute the stem cell population critical for perpetuating leukemia [45]. The involvement of K_V_11.1 in the physiology of leukemic and stem cells is further discussed in Section 4.4.

Overall, relatively ample evidence indicates that K_V_ channels are important modulators of normal and leukemic HSCs. It is, however, clear that the physiological comprehension of these processes is still in its infancy.

### 3.2. Neuronal Nicotinic Receptors (nAChRs) and Hematopoiesis

The neuronal nicotinic acetylcholine receptors (nAChRs) are ligand-gated pentameric ion channels that mediate fast excitatory postsynaptic potentials as well as slower paracrine actions of ACh. Growing evidence shows that nAChRs are also widely expressed in nonnervous tissue, including lymphocytes, and in cancer cells [46]. Since nicotine is a well-known risk factor for cancer, these observations have stimulated work aimed to determine whether and how nAChRs contribute to regulate the main cellular events associated with neoplastic progression, that is, cell proliferation and survival, invasiveness, the epithelial-mesenchymal transition and angiogenesis [47]. The nAChRs are homo- or hetero-pentamers of *α* (*α*2–*α*9) and *β* (*β*2–*β*4) subunits, arranged with different possible stoichiometries, characterized by different physiological and pharmacological properties [46, 47]. Interestingly, recent genomewide association studies attribute to the gene cluster coding for the *α*3/*α*5/*β*4 nAChR subunits a role in both development of lung cancer and nicotine addiction [48–50]. Many cellular effects of nicotine-and tobacco-derived metabolites are probably caused by nAChR activation, which leads to increased cytosolic calcium. This can stimulate intracellular pathways both directly as well as by increasing the release of autocrine/paracrine factors [51–53]. Nonconductive signals exerted by nAChRs have also been observed [54]. In general, the precise function of nAChRs in normal and neoplastic cells is difficult to determine because many cells express a variety of nicotinic subunits, whose interplay is unclear [47].

In humans, smoking is associated with increased leukocyte count [55–57]. Recent work in mice indicates that treatment with nicotine increases leukocytes in peripheral blood, BM, and spleen [58], in keeping with previous results showing that nicotine stimulates hematopoiesis [59]. The effect is correlated with higher frequency of HSCs in bone marrow [58]. Long-term HSCs isolated from treated mice appear to remain fully competent and express nAChRs, as demonstrated by binding of *α*-bungarotoxin (specific for *α*7-containing nAChRs and the muscle isoform). The expression pattern of other nAChR subunits was not reported. Regardless, these results are consistent with the previous observation that mice deficient of *α*7 display a reduction of myeloid and erythroid lineages [60]. Although the mechanisms of nAChR implication in these processes are unclear, these studies suggest that the cholinergic system is implicated in regulating hematopoiesis. From a pathologic standpoint, some of the effects of prolonged tobacco use may thus be caused by nAChR targeting. However, it should be kept in mind that *α*7 receptors desensitize rapidly in the presence of agonists. Therefore, to understand their physiological role in hematopoiesis, it will be important to precisely determine how these effects depend on the concentration of nicotine and other tobacco-related compounds. Low doses may sustain steady state nAChR currents, although with low amplitude, whereas higher doses may produce strong channel desensitization. In fact, the available evidence, albeit fragmentary, indicates that high concentrations of nicotine produce inhibition of hematopoiesis, instead of stimulation [60]. Hence, it is possible that different smoking habits produce opposite effects on hematopoiesis.

## 4. Ion Channels in Mesenchymal Stem Cells

### 4.1. MSCs from Bone Marrow (BM-MSCs)

Although human MSCs have been used for several years in the investigation of cell therapy and differentiation [36, 61, 62], a coherent picture of the physiological functions of the different ion channels expressed in these cells is not available. Not surprisingly, most results concern human BM-MSCs. In these cells, Kawano et al. first observed spontaneous inositol 1,4,5-trisphosphate-dependent Ca^2+^ oscillations, which are regulated by the Na^+^-Ca^2+^ exchanger and the plasma membrane Ca^2+^ pump [63, 64]. For review of the calcium handling system in MSCs, see [65]. In addition, approximately 10–15% of BM-MSCs express the L-type calcium current (*I*_CaL_) and related mRNAs such as **α*1C* [34, 35, 66], although *I*_CaL_ does not seem to be significantly implicated in controlling the spontaneous cell activity [66]. Nonetheless, the calcium oscillations are completely blocked by removing extracellular calcium and by applying La^3+^, while the intracellular stores are not depleted. This suggests that an unknown pathway, probably mediated by nonselective cation channels, controls calcium entry in hMSC and contributes to sustain the [Ca^2+^]_i_ oscillations [64]. These latter drive the activity of high-conductance Ca^2+^-dependent K^+^ channels (BK_Ca_), which in turn determines *V*_*m*_ cycling [64, 67]. In human BM-MSCs, K_Ca_ currents and the corresponding *MaxiK* mRNA were also observed by others, along with a slowly activating K^+^ current distinct from the rapidly activating K_Ca_ [34]. Significant mRNA expression was also detected for *K*_*V*_ *1.4*, *K*_*V*_ *4.2*, *K*_*V*_ *4.3*, and *HCN2*, but no corresponding functional currents were reported [34]. These were instead reported by Li et al. [35] who, besides BK_Ca_ and *I*_CaL_, measured transient outward voltage-dependent K^+^ channels sensitive to 4-aminopyridine whose molecular correlates were identified as *K*_*V*_ *1.4* and *K*_*V*_ *4.2 *and denoted in the literature as *I*_A_ or *I*_TO_ (for a brief summary of *I*_K_ nomenclature, see [68]). These authors also observed expression of K_V_10.1-delayed rectifying K^+^ channels (also known as ether-à-go-go type 1, EAG1), accompanied by the corresponding mRNA [35]. Finally, in hBM-MSCs, expression of K_ATP_ channels (Kir6.1 and Kir6.2) and the regulatory subunit SUR2A was detected by RT-PCR and immunolabeling. Osteogenic differentiation strongly increased Kir6.2, whereas adipogenic differentiation reduced Kir6.1 and SUR2A [69].

Broadly speaking, the above pattern seems also to apply to BM-MSCs prepared from rats [70–72] and mice [73, 74], where the effects on proliferation of modulating ion channels have been studied in some more detail. Rat BM-MSCs express both high- and intermediate-conductance K_Ca_ (resp., *slo* or *K_Ca_1.1* and *KCNN4 or K*_*Ca*_ *3.1*), *I*_KDR_ (*K*_*V*_ *1.2 and K*_*V*_ *2.1*), *I*_A_ (*K*_*V*_ *1.4* and *K*_*V*_ *4.3*), *I*_CaL_ and TTX-sensitive Na^+^ currents [70–72]. In these cells, K_DR_-decreased whereas intermediate-conductance K_Ca_3.1 increased during progression from G1 to S phase. Downregulating these channels with specific interfering RNAs blocked cell proliferation [71]. The partial substitution of K_DR_ with K_Ca_ during cell cycle progression agrees with the model originally proposed in T lymphocytes, in which sequential activation of K_V_ and K_Ca_ during mitosis triggers and sustains cell hyperpolarization, with ensuing facilitation of Ca^2+^ entry and stimulation of the cell cycle machinery [75]. In murine MSCs, the usual K_Ca_3.1 channel is accompanied by K_IR_ currents (Kir2.1) and volume-regulated Cl^−^ channels (CLCN3 [73]). K_Ca_ and Cl^−^ channels cooperate in regulating cell proliferation by modulating cyclin D1 and cyclin E expression [74]. K_Ca_, *I*_CaL_, and *I*_A_ (*K*_*V*_ *1.4*) were also observed in undifferentiated MSCs isolated from chicken embryos [76].

As is often the case, some types of K^+^ channels are involved in processes related to cell adhesion and migration. For instance, Hu et al. [77] found that K_V_2.1 regulates directed migration and homing of BM-MSCs. Hypoxic preconditioning increases K_V_2.1 expression and enhances the channel activity that subsequently augments phosphorylation/activation of the focal adhesion kinase and cell migration, resulting in increased homing of transplanted BM-MSCs to the injured region [77].

Finally, about 30% of human BM-MSC cells were found to express TTX-sensitive voltage-gated Na^+^ currents [34]. This observation was also carried out in rodents [70, 73] and chicken embryo MSCs [76], but its physiological meaning is uncertain.

### 4.2. MSCs from Human Umbilical Cord Vein (hUC-MSCs)

The expression of ion channels has been recently studied in undifferentiated hUC-MSCs [78]. Patch-clamp experiments revealed iberiotoxin-sensitive *I*_KCa_, *I*_A_, and *I*_DR_ currents. Once again, about 30% of these cells express functional TTX-sensitive Na^+^ currents, whereas no more than 5% of the cells express K_IR_. The molecular correlates of these currents were investigated by RT-PCR, which revealed the expression of *K*_*V*_ *1.1*, *K*_*V*_ *4.2*, *K*_*V*_ *1.4*, *Kir2.1*, *heag1*, *MaxiK*, *hNE-Na*, and *TWIK-1* [78]. Therefore, the overall pattern is similar to the one shown by BM-derived cells.

### 4.3. MSCs Derived from Human-Induced Pluripotent Stem Cells (iPSCs)

iPSCs produce a high yield of MSCs. Moreover iPSC-derived MSCs present higher proliferation capacity than BM-derived stem cells, thus providing a better option for applications in regenerative medicine. Zhang et al. [79] recently studied whether the different proliferative potential of iPSC-MSCs may be founded on significant differences in ion channel expression compared to BM-MSCs. Patch-clamp measurements revealed five functional ion currents in human iPSC-MSCs: BK_Ca_, intermediate-conductance K_Ca_, K_DR_, K_IR_, and voltage-gated Cl^−^ channels, whereas the latter Cl^−^ currents were not detected in BM-MSCs. RT-PCR revealed significant expression in both cell types of *K*_*Ca*_ *1.1, K*_*Ca*_ *3.1, K*_*V*_ *10.1, Kir2.1, SCN9A, CACNA1C,* and *Clcn3*. In contrast, *Kir2.2* and *Kir2.3* were only found in iPSC-MSCs. Interestingly, the expression level of *K*_*V*_ *10.1* was much higher in iPSC-MSCs than in BM-MSCs. In agreement with the proliferative potential conferred by K_V_10.1 (hEAG1) to different cell types [80], block of hEAG1 tended to inhibit cell proliferation in both cell types. Consistently with the expression pattern, the effect was more pronounced in iPSC-MSCs.

These observations may contribute to explain the greater proliferative capacity of iPSC-MSCs, which may be correlated with specific patterns of channel expression. In general, the diversity of ion channel expression in MSCs might reflect the existence of different cell populations. Differently from immortalized cell lines, pure populations of MSCs have been so far impossible to isolate. As discussed earlier, identification of human MSCs is mainly based on their plastic-adherence features in standard culture conditions and expression or lack of expression of an ensemble of surface markers. As has been observed in other cell types, some channel types seem specifically implicated in controlling cell cycle. Interestingly, evidence summarized in the above sections also suggests that K_IR_ currents tend to be expressed in stem cells at earlier stages, that is, in cells with broader differentiation potential. This may be different from what has been observed in the nervous system, where for example, K_IR_ currents mark late stages of quail neural crest cell differentiation [81].

### 4.4. K_V_11.1 (hERG1) Channels in Leukemic Cells Mediate Cell Interaction with MSCs

BM-MSCs can protect leukemic cells from chemotherapy by secreting the chemokine SDF-1 (also known as CXCL11), which binds to the G-protein-coupled receptor CXCR4 (chemokine receptor CXC 4 [82]) expressed onto leukemia cells [83]. Adhesion between these cell types is consolidated by engagement of the integrin receptors expressed onto leukemic cells, typically *α*5*β*1 (VLA-5) and *α*4*β*1 (VLA-4). These interact with extracellular matrix proteins on the MSCs [84]. MSCs thus regulate intracellular signaling cascades in leukemic cells that lead to antiapoptotic effects, as shown in acute myeloid leukemia [85], chronic lymphocytic leukemia [86–88], and chronic myeloid leukemia [89]. These intracellular signals are thought to require integrin-dependent activation of ILK (integrin-linked kinase), with subsequent recruitment of the MAPK and the phosphoinositide 3-kinase (PI3K)/Akt pathways, at least in myeloid leukemia.

We recently studied the molecular mechanisms underlying the analogous effect observed in acute lymphoblastic leukemia (ALL) cells [90]. Coculture of ALL cells with MSCs induced on the lymphoblast plasma membrane the expression of a signaling complex formed by hERG1, the *β*1 integrin subunit, and CXCR4. Such complex is absent in normal B lymphocytes. Moreover MSCs also do not express hERG1. Assembly of the protein complex activated the prosurvival pathways centered on ILK, extracellular signal-related kinase 1/2 (ERK1/2) and PI3K/Akt. In parallel, ALL cells became resistant to chemotherapy-induced apoptosis [90]. Formation of an hERG1/integrin/VEGF receptor complex was previously observed in acute myeloid leukemia [19]. The possibility that interaction with CXCR4 also occurs in these cells is matter for future studies.

Blocking hERG1 channels with specific drugs decreased the protective effect conferred by MSCs to leukemic cells function. Moreover, integrin activation depends on channel activity. The channel role appears to be important for both initiation of pro-survival signals and development of drug resistance. Immunodeficient mice engrafted with ALL cells and treated with channel blockers had increased rate of leukemic cell apoptosis, reduced leukemic infiltration and overall survival rates. What is more, hERG1 blockade enhanced the therapeutic effect produced by corticosteroids.

### 4.5. MSCs and Nicotinic Receptors

Observations such as the relatively recent one that smoking cessation in chronic smokers produces a rapid increase of circulating endothelial progenitor cells in peripheral blood led to suspect that the tobacco products regulate MSC physiology [91]. In fact, it was subsequently found that human MSCs express the entire machinery for cholinergic signaling, namely, the synthesizing enzyme choline acetyltransferase, the degrading enzyme acetylcholinesterase, ACh itself, the nAChR subunits *α*3, *α*5, and *α*7 and the muscarinic ACh receptor M2 [92]. Human MSCs were also found to express *β*2 and *β*4 nAChR subunits [93]. Nicotinic and muscarinic receptors are expressed in different MSC populations [92], but stimulation with receptors' agonists leads to a rapid increase of intracellular (Ca^2+^) in both [92, 93]. As in other cell types [47], this signal converges onto ERK1/2 [92]. These results are consistent with the general notion that nonneuronal tissues can express the entire cholinergic machinery necessary to release ACh for paracrine signaling. Tonic exposure to nicotinic ligands in smokers alters proper regulation of such system. Interestingly, *α*7 nAChRs were found to be implicated in the control of MSC migration [93], in analogy with what was previously observed in human keratinocytes [94]. The balance of the nAChR effects on proliferation/apoptosis and migration is a matter for future studies.

## 5. Conclusion

Knowledge about the cell physiology of ion channels and transporters in stem cells is at an initial stage. Current evidence indicates that, in analogy with what has been observed in other cell types, stem cells, particularly the better known human MSCs, express a wide variety of ion channels which are implicated in different physiological functions. Broadly speaking, certain classes of K^+^ channels cooperate with Ca^2+^ and Cl^−^ channels in regulating the calcium transients and the volume oscillations that accompany the cell cycle. Other K^+^ channel types control cell anchorage with the stromal matrix and cell migration as well as release of paracrine growth factors. The differential expression of ion channel types in individual MSC cells indicates that stem cell populations may be heterogeneous. One possibility is that, as observed in other cell types, MSCs sampled at different cell cycle phases present different channel activity or expression (reviewed in [8]). Another possible explanation is that MSC populations contain progenitor cells at different stages of commitment, which would also affect the pattern of ion channel expression and activity. Recent evidence in both HSCs and MSCs indicates that ligand-gated channels, such as nAChRs, also contribute to regulate stem cell biology.

From a therapeutic standpoint, MSCs have been lately the object of intense interest. The perspectives these cells offer for clinical applications turn on their easy accessibility, prompt expansibility, and capacity to differentiate. A more thorough understanding of their physiological features, including the details of how different ion channels and transporters interplay, will be necessary for safer and reliable medical applications. Currently, four main therapeutic approaches are being investigated: (i) local implantation of MSC for focalized diseases, (ii) systemic transplantation, (iii) combination of stem cell therapy with gene therapy, and (iv) use of MSC in tissue repair and remodeling. Considering the advantages offered by membrane channels as targets of pharmacological therapy [95], we believe further efforts along this line should suggest potentially fertile novel methods to attempt therapy in both normal and cancer stem cells.
